# A critique of the crowd psychological heritage in early sociology, classic phenomenology and recent social psychology

**DOI:** 10.1007/s11007-022-09566-z

**Published:** 2022-03-14

**Authors:** Gerhard Thonhauser

**Affiliations:** Department of Philosophy, TU Darmstadt, Karolinenplatz 5, 64289 Darmstadt, Germany

**Keywords:** Crowd psychology, Emotional contagion, Gustave Le Bon, Max Scheler, Max Weber, Georg Simmel

## Abstract

The paper critically reconstructs the crowd psychological heritage in phenomenological and social science emotion research. It shows how the founding figures of phenomenology and sociology uncritically adopted Le Bon’s crowd psychological imagery as well as what I suggest calling the disease model of emotion transfer. Against this background, it can be examined how Le Bon’s understanding of emotional contagion as an automatic, involuntary, and uncontrollable mechanism has remained a dominant force in emotion research until today. However, a closer look at phenomenological descriptions and empirical investigations of how emotion’s spread shows that there is little evidence supporting Le Bon’s crowd psychological framework. Thus, I suggest that the disease model should be dismissed in favor of more plausible approaches to interpersonal emotion dynamics.

## Introduction

The aim of this paper is to critically reconstruct the heritage of Le Bon’s crowd psychological framework in phenomenological and social science research on emotional processes in gatherings of various sizes. This heritage mainly consists of two interconnected features: First, the crowd psychological imagery of the crowd as an animalistic, filthy, and violent social unit, which implies that crowds are beyond rational control and thus of dubious moral status and low social value. Second, the influence of what I suggest calling the disease model of emotion transfer, implying that emotions are transmitted between individuals beyond conscious awareness and control.

This paper applies a double strategy: On the one hand, I show how major figures in early sociology, classic phenomenology, and more recent social psychology adopted Le Bon’s crowd psychological framework – either rather uncritically or without awareness thereof. It is impossible, of course, to investigate the entire history of 20th and 21st century emotion research to prove this point, not least within one research paper. However, by focusing on three paradigmatic contexts which are crucial for the development of sociology, phenomenology, and social psychology respectively, and which are prominent sites of Le Bon’s influence, I am confident that the paper can provide some plausibility for this first conclusion.

On the other hand, I support two claims: First, the concept of the “crowd,” as Le Bon defined it, does not correspond to an actual phenomenon. Second, the concept of “emotional contagion,” again as defined by Le Bon, misconceives what is going on when individuals emotionally affect each other. An extensive defense of those claims goes beyond the scope of one paper. But luckily, the paper can build on existing literature. At least two book-length studies have argued for the implausibility of Le Bon’s notion of the crowd.^1^ This paper can be read as a small contribution to further cement this first claim. Regarding the issue of emotional contagion, there is a lively debate in current social psychology which will be discussed in the last section. This paper provides historical depth to that debate.

I proceed in four steps. First, I present an account of how crowd psychology was founded at the end of the 19th century by Le Bon. This is crucial for understanding the ideological background from which the crowd psychological framework emerged and how this informed the imagery on which it was based. Second, the paper looks at the founding figures of French and German sociology to show how they related to the crowd psychological framework. For the French context, I briefly reconstruct the alternative between the frameworks of Durkheim and Tarde as potential foundations of French sociology, which was quickly decided in favor of Durkheim. Then, I show how Simmel and Weber laid the foundations of German sociology by excluding the issue of the crowd from the subject area of sociological research. Third, I look at Scheler as one of the founding fathers of phenomenology and arguably the most influential voice concerning the understanding of emotions and particularly their potential sharedness within classic phenomenology. I show how Scheler’s seminal understanding of emotional contagion and its distinction from other forms of emotional sharedness is strongly influenced by the crowd psychological framework. Finally, I turn to the mimicry model of emotional contagion, which was introduced by Hatfield, Cacioppo, and Rapson and remains prominent in current social psychological emotion research. This allows me to examine how a crowd psychological understanding of emotional contagion as an automatic, involuntary, and uncontrollable mechanism remains a dominant force in today’s emotion research, even when that heritage is not explicitly noted as such.

Sections 2 and 3 provide more of a historical reconstruction of the crowd psychological framework with its imagery of the crowd and the disease model of emotion transfer. Sections 4 and 5 offer a more systematic discussion of the issues at hand. Together they lead to my concluding recommendation that we should actively avoid relapsing into the imagery of the crowd and the disease model of emotion spreading for the benefit of more plausible approaches to the study of social groups and emotional dynamics in interpersonal contexts.

One final introductory remark: The following investigation is based on an examination of the original German and French sources. In some instances, English editions became available only long after the period investigated here. In other instances, English translations hide the crowd psychological traces, and more importantly, conceal the problematic ideological import of crowd psychology. Thus, one needs to go back to the original sources to trace the influence of the crowd psychological framework.

## Le Bon’s introduction of crowd psychology

This section offers a brief history of how crowd psychology originated from France at the end of the 19th century. The founders of crowd psychology are Gabriel Tarde and, most importantly, Gustave Le Bon. Both rely on Hippolyte Taine’s *Les Origines de la France Contemporaine*.^2^ A brief look at this work provides an insight into the ideological context from which crowd psychology emerged.^3^ Taine’s work offers a history of France’s many revolutions over the course of the 19th century. It is written from the perspective of a bourgeois who looks down on the revolting masses, considering their behavior as filthy and bestial. From Taine’s point of view, the crowd is a threat to the natural and good order of society, which he identifies as a hierarchical order in which few are chosen to rule. By contrast, he thinks that democratic government will necessarily end in violence and destruction.

As we will see in the following section, Tarde’s goals were primarily theoretical – aiming at a methodological foundation of sociological research – which makes it plausible to detach his work from the ideological connotations of its historical origin. By contrast, Le Bon not only embraced Taine’s political orientation, but offered a much more radicalized political outlook. Le Bon was an outspoken elitist and racist. He was convinced that each “race” has a particular “soul” and that those souls stand in a natural hierarchy. Analogously, he claims that there is a natural hierarchy and order in each society according to which some are born to rule and others are born to follow. Le Bon’s most well-known book, *Psychologie des Foules* (literally translated: *Psychology of the Crowd*), is part of a trilogy: One year prior to its publication, Le Bon published *Les Lois Psychologiques de l’Évolution des Peuples* (translated as *The Psychology of Peoples*), one year later *Psychologie du Socialisme* (translated as *The Psychology of Socialism*).^4^ This trilogy reflects Le Bon’s three key enemies: other “races,” lower classes, and socialism. The first two volumes are meant to provide “scientific” proofs for his racist claim and his elitist claim. Le Bon sees socialism as an enemy because he understands its claim for equality to be a subversion of the natural order of society.

The English edition hides how *The Crowd* is embedded within this trilogy. In the French original the first two sentences of *Psychologie des Foules* read as follows: “Notre précédent ouvrage a été consacré à décrire l’âme des races. Nous allons étudier maintenant l’âme des foules.”^5^ The English translation omits the entire first sentence with its reference to Le Bon’s previous work on the “characteristics of races” (obviously, Le Bon refers to *The Psychology of Peoples*) and starts with the innocuous second sentence, “The following work is devoted to an account of the characteristics of crowds.”^6^ However, the second paragraph of *The Crowd* makes the connection clear. It summarizes Le Bon’s racist claim from the previous book, according to which each “race” has its own “soul” (*l’âme*), that is, certain essential features “which heredity endows the individuals of a race.”^7^ As can be studied in detail in his previous work on *The Psychology of Peoples*, Le Bon advocates a hierarchy of “races” based on biological determinism.^8^ This openly racist origin of Le Bon’s work is often ignored in discussions of crowd psychology.

In the preface of *The Crowd*, Le Bon asserts that he addresses the issue of the crowd “in a purely scientific manner.”^9^ However, this statement is misleading and deceptive. To begin with, Le Bon’s entire writing is popular scientific, which might also explain its popular success, as Le Bon aimed to address a broader audience. Le Bon does not present extended arguments, but rather works by persuasion. The lack of arguments in his texts means that there is little that can be refuted by means of philosophical reasoning. Rather, a critique of Le Bon’s work must primarily proceed by exposing the imagery with help of which it aims to achieve persuasiveness. Le Bon’s entire project is motivated and justified by his racist and elitist convictions, which is conveyed in his selection of words and metaphors.

From the outset, Le Bon aims at the slandering and maligning of the “crowd,” which is his term for the alleged soul of the plebs. This is already indicated by the choice of the term “*foule*.” To describe a gathering as a “*foule*” already points towards evaluating it as ignorant and potentially out of control. In everyday language, the word is used for spectators of a football match or a group of protesters, but not for a theatre audience or a group of law enforcement officials. The same can be said for the German term “*Masse*,” which is used as the translation of “*foule*” in the German edition of Le Bon’s work and sparked the debate under the label “*Massenpsychologie*” (see the following two sections). Note, however, that Le Bon also considers juries or parliaments as examples of crowds.^10^ This is another sign of his elitist view on society. As a 19th century conservative believing in natural hierarchies, Le Bon strongly opposed popular participation in politics (he championed *governo stretto* over *governo largo*, one could say with Machiavelli). Thus, he saw participatory institutions like parliaments and juries as signs of a decay from the natural order. Building on the connotations of the term “*foule*” in everyday language, the imagery of Le Bon’s text focuses on the connection of the crowd with animality, barbarism, and filthiness. He characterizes crowds as irrational and irresponsible, displaying a propensity to madness and violence.^11^ He defines their influence as always of a destructive kind: “Civilisations as yet have only been created and directed by a small intellectual aristocracy, never by crowds. Crowds are only powerful for destruction.”^12^

In chapter 1 of *The Crowd* Le Bon defines what he considers “the mind of the crowd.” This contains the substance of Le Bon’s theory. Le Bon’s approach is crowd psychological in the strict sense of the word: He aims at a psychological understanding of the crowd. According to Le Bon’s definition, the crowd is a social unity with a mind of its own:

In its ordinary sense the word ‘crowd’ means a gathering of individuals of whatever nationality, profession, or sex, and whatever be that chances that have brought them together. From the psychological point of view the expression ‘crowd’ assumes quite a different signification. Under certain given circumstances, and only under those circumstances, an agglomeration of men presents new characteristics very different from those of the individuals composing it. The sentiments and ideas of all the persons in the gathering take one and the same direction, and their conscious personality vanishes. A collective mind is formed, doubtless transitory, but presenting very clearly defined characteristics. The gathering has thus become what, in the absence of a better expression, I will call an organised crowd, or, if the term is considered preferable, a psychological crowd. It forms a single being, and is subjected to the *law of the mental unity of crowds*.”^13^

As this definition makes clear, not all gatherings are crowds in Le Bon’s psychological sense, but only those that are constituted by this supposed crowd mind. And crowds are not restricted to large gatherings, also small groups can be crowds in Le Bon’s sense. Becoming part of a crowd implies a loss of individuality and a fusion into the collective mind of the crowd. This “collective mind […] is entirely dominated by unconscious elements, and is subject to a peculiar collective logic.”^14^ Crucially in the eyes of Le Bon, “it is stupidity and not mother-wit that is accumulated” in the collective mind of the crowd.^15^ Le Bon states that “by the mere fact that he forms part of an organised crowd, a man descends several rungs in the latter of civilisation.”^16^ This imagery of the crowd as barbarian, filthy, and potentially violent is all that constitutes the alleged persuasiveness of Le Bon’s crowd psychology.

Le Bon lists three mechanisms that lead to the emergence of this supposed crowd mind. First, the crowd is governed by sentiment instead of reason, with the sentiment of the crowd being so powerful that it overrides individual reasoning.^17^ Here Le Bon makes use of the classic trope of a sharp distinction between reason and emotion, claiming that the presence of emotions inhibits the reasoning capacities of individuals. Moreover, he seems to imply that the ability to reason is linked with individual minds, whereas a crowd is defined by the presence of collective sentiments that override individual reasoning. In this paper, I focus on the second mechanism listed by Le Bon – contagion – as this concept was the most impactful. The term *contagion* is meant to invoke the idea of an infectious disease. Le Bon defines contagion as the instance of a sentiment, thought, or wish being transmitted from one person to another without an awareness of that transfer and without any possibility of conscious control.^18^ In the case of an infection, it does not make sense to ask for reasons. One can only identify causal mechanisms that have led to an infection. If one wants to understand why one has become ill, one needs to trace the source of the infection. This can only be done retrospectively. In the moment in which the infection is happening, there is no way to voluntarily intervene and resist the infection. One can only anticipate potential risks and avoid dangerous situations (like crowded places or sick people). In short, disease transmission happens unintentionally, uncontrollably, and unnoticedly, and only allows to ask for causes, not for justifications. The third mechanism identified by Le Bon is suggestibility, which means the state of openness which makes an individual susceptible for contagion. Le Bon compares suggestibility to being hypnotized, which he defines as a state in which “the conscious personality has entirely vanished; will and discernment are lost. All feelings and thoughts are bent in the direction determined by the hypnotiser.”^19^

Hopefully, the remarks in this section have been sufficient to expose the racist and elitist origins of Le Bon’s thought and the platitude of his argument. The following sections show how Le Bon nevertheless managed to considerably influence 20th century thought.

## The influence of crowd psychology on the foundation of sociology

As I already mentioned in the previous section, Le Bon’s work on the crowd was influenced by Gabriel Tarde’s *The Laws of Imitation*, which was published in 1890, five years prior to *The Crowd*.^20^ For the English audience, however, the order of publication was reversed, as Le Bon’s book was already translated in 1896, while Tarde’s book first appeared in 1903.^21^ This shows that Le Bon’s popular scientific writing introduced crowd psychology to an international audience. This is even more so the case in the German context. The German reception of Le Bon was a bit slower compared to the US, with the first German edition appearing in 1908.^22^ However, Tarde’s work was not translated until the 21st century, which means that the German debate on *Massenpsychologie* was almost exclusively based on Le Bon’s writing.^23^

In contrast to Le Bon’s ideological aims of maligning and controlling the supposedly maddening crowd, the objective of Tarde’s work was to found sociology as a scientific discipline. He wanted to present the general laws of the social that apply to all societies.^24^ His core claim is that “imitation” is the general mechanism on which all societies are based. (We will see shortly that “social interaction” plays a similar role for Simmel, as does “social action” for Weber). Tarde defines a society as “a group of people who display many resemblances produced either by imitation or by *counter-imitation*.”^25^ For the context of this paper, there is no need to study Tarde’s work in more depth, as it had virtually no influence on the developments we aim to dissect here. Regarding the foundation of sociology, Tarde was quickly forgotten to the benefit of his contemporary Emile Durkheim who became the main founder of sociology in France.^26^ In 1895, Durkheim published *Les Règles de la méthode sociologique* (*The Rules of Sociological Method*).^27^ In this book, he is concerned with defining the phenomena that are the distinct and exclusive concern of the new science of sociology. He finds those phenomena in what he calls “social facts,” which are large-scale constraints on the actions of individuals. This shows how Durkheim founded French sociology based on a holistic paradigm, which sets it apart from the methodological individualism dominant in German sociology (most prominently in the version of Weber). This holistic orientation allows a Durkheimian sociology to address collective phenomena otherwise associated with crowd psychology, whereas Weber’s methodological individualism leads him to exclude such phenomena from sociological research. For instance, Durkheim’s concept of “collective effervescence” addresses cases of collective emotion in which the connections within a gathering become so strong that individuals feel themselves as part of a bigger unity that overrides their individuality.^28^ In contrast to Le Bon’s crowd psychology, however, Durkheim’s work has the double advantage of being based on empirical research and forgoing an ideological evaluation of its research object.^29^

In contrast to the French context, where the Durkheimian holistic understanding of the social meant that crowd psychology was contested by a more plausible framework, the methodological individualism of German sociology entailed that the topic of the alleged crowd was excluded from sociological concerns. This had the odd consequence that the crowd psychological framework was tacitly accepted by sociologists as the appropriate approach for the investigation of large gatherings. A first instance of this argumentative move can be found in Simmel’s 1908 book *Soziologie: Untersuchungen über die Formen der Vergesellschaftung* (*Sociology: Inquiries into the Construction of Social Forms*).^30^ Simmel aims at founding sociology on the “insight that the human being may be defined in all its essence and manifestations as living in interaction with other human beings.”^31^ Simmel considers “interaction” as the key concept defining the subject area of sociology, stating that “a society exists where several individuals enter into interaction.”^32^ According to Simmel, this is true from the smallest to the largest groups. However, regarding larger gatherings he presents the psychology of the crowd as an exception to the laws of society investigated by sociology: “As soon as great masses are set into motion […] they display a thoughtless radicalism. […] Here the ebb and flow of countless suggestions produce an extraordinarily strong nervous excitement that often carries the individuals along unconsciously; every impulse swells up avalange-like, and allows the crowd to become the prey of the ever most passionate personality in it.”^33^ Simmel repeats this definition of crowd behavior throughout the text, hence showing how he fully accepts the crowd psychological approach to such phenomena.

The argumentative move to delineate the subject matter of sociology by distinguishing it from the issue of crowd psychology is even more pronounced in Max Weber’s *Economy and Society*, another classic of German sociology.^34^ In § 1 Weber presents his definition of sociology, which has become formative for the discipline, at least in Germany. According to Weber, sociology is concerned with *social action.* Action is defined here as behavior to which the acting individual attaches a subjective meaning. Social action implies that this meaning takes the behavior of others into account.^35^ According to Weber’s methodological individualism, the social needs to be understood based on the actions of individuals. However, Weber mentions an exception to that rule: “It is well known that the actions of the individual are strongly influenced by the mere fact that he is a member of a crowd confined within a limited space. Thus, the subject matter of studies of ‘crowd psychology,’ such as those of Le Bon, will be called ‘action conditioned by crowds.’”^36^ For Weber’s foundation of sociology, it is decisive that one sharply distinguishes between *social action* and *action conditioned by crowds*, and thus, between the subject matters of sociology and crowd psychology. At the same time, Weber acknowledges that it might be difficult or maybe impossible to empirically draw this distinction.^37^ But more importantly, Weber’s distinction implies that he accepts Le Bon’s characterization of the crowd and Crowed-based behavior. This had the ominous consequence of making sociology blind for certain collective phenomena in large gatherings, as they were simply ruled out as a worthy subject of sociological research. One example are sports audiences, which have long been an unpopular topic of research in German sociology, as they were considered an example of the filthiness of the crowd.^38^

## The Influence of crowd psychology on Scheler’s foundation of phenomenology

Around the same time as Simmel and Weber laid the groundwork for German sociology, Max Scheler set the stage for the way in which classic phenomenology approached sociality. Most importantly, Scheler adopted the distinction of community (*Gemeinschaft*) and society (*Gesellschaft*), which was introduced by Ferdinand Tönnies (another founding father of German sociology) and supplemented it with the notion of the crowd (*Masse*) from crowd psychology.^39^ Scheler connected this taxonomy of social units (crowd, community, society) with the question of how the different social units relate to different forms of what Scheler calls “fellow-feeling” (*Mitgefühl*).^40^ Scheler’s taxonomy of social units and his focus on their connection to the forms of fellow-feeling shaped the way in which classic phenomenology discussed sociality. There is disagreement among Scheler scholars how the different social units precisely relate to the forms of “fellow-feeling.”^41^ However, that issue does not need to concern us here. Instead, I will focus on uncovering the influence of Le Bon’s crowd psychology on Scheler’s taxonomies.

But before we dive into the content of Scheler’s work, it is important to consider the publication history of the relevant texts. The book which is known to English-speaking readers under the title *The Nature of Sympathy* first appeared in 1913 as *Zur Phänomenologie und Theorie der Sympathiegefühle und von Liebe und Hass* (literally translated: On *the Phenomenology and Theory of Sympathetic Feelings and of Love and Hate*).^42^ The same year, the first volume of Scheler’s major work *Formalism in Ethics and Non-Formal Ethics of Values* appeared, with a second volume following in 1916.^43^ Ten years later, *The Nature of Sympathy* saw a second edition with the title *Wesen und Formen der Sympathie* (literally translated: *Essence and Forms of Sympathy*).^44^ This second edition forms the basis for all English translation. However, if one wants to study the influence of Le Bon’s work, one should focus on the first edition in its connection to the simultaneously written *Formalism in Ethics*.

Scheler introduces his taxonomy of possible social units towards the end of *Formalism in Ethics*. His very short definition of the crowd reveals how Scheler follows Le Bon’s imagery:

A social unit is constituted (simultaneously) in so-called contagion and involuntary imitation devoid of understanding. Such a unit of animals is called the herd, of men, the mass. With respect to its members, the mass possesses a reality of its own and has its own laws of effectiveness.”^45^

Let me dissect this definition sentence by sentence. Beginning from the end, the third sentence alludes to the claim that a crowd has a mind of its own, which is the key systematic claim of Le Bon’s crowd psychological framework. The second sentence draws an analogy between crowds and animal herds, which is also very much in line with Le Bon’s imagery. In a remark added to the second edition of *The Nature of Sympathy* Scheler states that, “man becomes more of an animal by associating himself with the crowd.”^46^ Finally, the first sentence states that the crowd (*Masse*) is constituted by contagion (*Ansteckung*) and imitation (*Nachahmung*). As the source for this claim Scheler refers to his own work *The Nature of Sympathy*. In the relevant passage from *The Nature of Sympathy*, Scheler refers to two books by Le Bon, *Psychologie des Foules* and a book with the title “*L’âme révolutionnaire*.”^47^ Only in the second edition from 1923, Scheler significantly extends the footnote, adding references to Tarde’s *Les Lois de l’imitation* and Sigmund Freud’s *Group Psychology and the Analysis of the Ego* from 1921.^48^ With “*L’âme révolutionnaire*,” Scheler most likely refers to Le Bon’s book *La Révolution Française et la Psychologie des Révolutions* from 1912.^49^ When one compares Le Bon’s characterization of the crowd in this book (which is a condensed summary of *The Crowd*) with Scheler’s definition one finds a very strong, almost verbatim correspondence.^50^

The cornerstone of Scheler’s adaptation of crowd psychology is the notion of contagion in *The Nature of Sympathy*. Let me prepare an examination of the crucial passages by briefly introducing Scheler’s taxonomy of “fellow-feeling” (*Mitgefühl*). Scheler begins his characterization by stating that fellow-feeling needs to be distinguished from all behavior which “merely contribute to our *apprehending*, *understanding*, and, in general *reproducing* (emotionally) the experiences of others, including their states of feeling.”^51^ For Scheler, apprehending (*Nachfühlen*) an other’s emotion is a precondition and a component of fellow-feeling, but not in itself a form of fellow-feeling.^52^ This is the case because fellow-feeling requires that one has a feeling of one’s own, whereas I can apprehend the feelings of others without any emotional reaction. After this initial distinction, Scheler differentiates three phenomena: “immediate community of feeling” (*das unmittelbare Mitfühlen*); “fellow-feeling ‘about something’” (*Mitgefühl an Etwas*); and “mere emotional infection” (*bloße Gefühlsansteckung*).^53^ (In the second edition Scheler adds the category of “emotional identification” (*Einsfühlung*), but this does not need to concern us here.^54^) First, “community of feeling,” Scheler also calls it “feeling-in-common” (*Miteinanderfühlen*), is defined as two or more individuals sharing the same feeling; there feeling is “*one and identical.*”^55^ There is a debate among Scheler scholars about how exactly one should understand this claim. Most importantly, the sameness in Scheler’s example oscillates between token- and type-identity.^56^ However, those details are also not relevant in the context of this paper. Second, fellow-feeling “about something” is different from feeling-in-common as it “involves *intentional reference* […] to the other person’s experience.”^57^ Hence, in this type of fellow-feeling individuals do not have the same feeling, but rather, one individual apprehends the feeling of another, and based on that apprehension, emotionally responds to the other’s feeling. Finally, Scheler discusses emotional contagion or infection (both terms serve as translations of *Gefühlsansteckung*). Scheler begins his elaboration on emotional contagion by emphasizing its difference from fellow-feeling. The defining difference is that fellow-feeling presupposes an apprehension of the other’s feeling, whereas contagion is precisely defined by the absence of such an apprehension.^58^ For that reason, contagion does not constitute a case of fellow-feeling for Scheler. Rather, his core claim is that others have confused contagion with fellow-feeling, and thus, failed to get a proper understanding of fellow-feeling.

Before discussing the distinction of fellow-feeling and contagion further, let me show how Scheler’s description of contagion relates to Le Bon’s definition. Scheler embraces Le Bon’s understanding of emotional contagion as a mechanism that seizes an individual beyond conscious awareness and control: The process is “involuntary” and “unconscious”; in a crowd it happens “like an avalanche,” leading to actions “for which no one acknowledges either the will or the responsibility.”^59^ Like in the case of Le Bon, the analogy of emotional contagion with the transmission of an infectious disease is guiding Scheler’s description. When we are infected by an emotion, the emotion does not involve any indication of its origin. Only retrospectively, via inferences and causal considerations, one can notice that one’s emotion is caused, for example, by one’s participation in a certain event.^60^ Accordingly, there is no way to resists infection through an act of the will, one can only attempt to avoid exposure.^61^

This analogy of emotion transfer and disease transmission is not only embraced by Scheler, but also by other classic phenomenologists. Classic phenomenologist followed Le Bon in assuming that (what I suggest calling) the disease model has a very broad applicability. It was taken to apply to all types of affective states, both with a positive and negative valence. Scheler’s examples include mourning, laughter, and joy.^62^ It was not bound to large gatherings but applied to groups of all sizes. And it was not restricted to human beings but also used in connection with other animals. Finally, not only emotions, but also thoughts, wishes, and other mental states were thought to be contagious. This is again in line with Le Bon, who speaks of “mental contagion” or “mental contagion and suggestion” in general.^63^ Hence, both within the crowd psychological framework and within classic phenomenology, this specific concept of contagion is considered to explain a wide range of phenomena.

What is at stake in the disease model can be made explicit with reference to Edith Stein. Stein, summarizing the common theorizing of her time, discusses *suggestion* as “the ‘implanting’ of ‘notions,’” which “means that the notion is taken over ‘without any logical reason.’”^64^ When becoming infected with a proposition, questions about its justification are suspended. One can only ask retrospectively about causal mechanisms that have led one to believing it. Stein offers the following example, which resonates with the elitist and anti-socialist sentiment guiding Le Bon’s crowd psychology: “If you wanted to designate Bolshevism today as an infectious disease of the psyche, then you’d mean (at least in general) that the ‘*ideas’* of Bolshevism transmit themselves like pathogenic agents from one individual to another and intrude upon him ‘suggestively.’”^65^ The message of this comparison of a political movement with an infectious disease is clear: One cannot support this movement based on justified reasons, but only based on an unwarranted acquisition of erroneous convictions.

An interesting question to ask is how one can distinguish contagion from fellow-feeling. The distinction between fellow-feeling in the narrow sense (fellow-feeling “about something”) and contagion is rather straightforward and unproblematic: The former presupposes an apprehension of the foreign feeling whereas the latter is defined by a lack of such an apprehension. More interesting is the distinction between contagion and feeling-in-common, a distinction that is still highly relevant in contemporary phenomenological accounts of collective emotions. Dan Zahavi, for instance, refers to Scheler when stating that emotional contagion needs to be sharply separated from emotional sharing.^66^ However, what exactly constitutes the distinction between contagion and feeling-in-common? In the case of feeling-in-common, Scheler claims that there is no apprehension of each other’s feeling, but an immediate feeling together. Thus, apprehension cannot serve as a criterion for deciding whether an emotional episode is a case of feeling-in-common or a case of contagion.

We have already encountered the main criterion in the passage from Edith Stein cited in the previous paragraph. In feeling-in-common, the emotion is justified, because it is motivated from within the individual having the emotion and answers to what is demanded by the situation. Consider Scheler’s famous example of the parents grieving over the death of their child.^67^ Obviously, the situation demands grief, and the grief is deeply motivated from the perspective of both parents. By contrast, when infected, the emotion comes from an external source and is not motivated from the perspective of the infected individual, and thus, it is deemed unjustified. However, how could one draw that distinction within one’s experience? For the entire point about contagion is that one does not notice that an emotion is caused by an external source, and therefore, mistakes it for one’s own emotion. Only retrospectively one might find out that one’s emotion was caused by contagion.^68^ Maybe one can argue that Scheler’s distinction has an analogous status as Weber’s distinction between social action and action conditioned by crowds. For both, the distinction serves as a crucial theoretical building block, but it might be difficult or maybe impossible to draw that distinction, not least empirically.

This can be exemplified with reference to one of Scheler’s examples. In the text *Der Krieg als Gesamterlebnis* (*The War as Total Experience*) from 1916, Scheler describes the German experience of World War I as a case of feeling-in-common.^69^ In the second edition of *The Nature of Sympathy* from 1923, however, he classified the same experience as a case of emotional contagion.^70^ In 1916, Scheler, like many German intellectuals, was in favor of the “Great War.” Seven years later, this enthusiasm appeared in a very different light. So Scheler retrospectively determined that the war enthusiasm he had felt a few years earlier was not a true feeling-in-common after all, because it was caused by infection. The examples of emotional contagion provided by Scheler and Stein fuel the suspicion that categorizing an emotional episode as caused by emotional contagion often serves the purpose of expressing a devaluation.^71^ To state it provocatively: One could not truly believe in the “Great War,” just as one cannot be a communist for justified reasons. Therefore, both convictions must be explained as the result of contagion.

But after all, I think that hidden beneath all the biases and prejudices, Scheler was up to something important. Scheler’s core systematic claim is that emotional contagion is not a form of fellow-feeling and should not be confused with it. According to my view, what is at stake here is the distinction between affective we-experiences and underlying psychological mechanisms.^72^ Scheler noticed this crucial distinction and pointed out how others had failed to account for it – he mentions Spencer, Schopenhauer, and Nietzsche, among many others.^73^ But in the end, the way in which he uncritically adopted the crowd psychological framework prevented him from developing the full potential of this finding.

## Traces of crowd psychology in recent social psychology

This final section concerns the way in which emotional contagion is conceptualized within current social psychology. In contrast to the early stages of sociology and classic phenomenology, there is no direct reference to Le Bon within this current research field. However, there are similarities to Le Bon’s crowd psychological framework which are worth examining. Moreover, newer developments in the field point towards discarding the remainders of the crowd psychological framework and moving towards better accounts of interpersonal emotion dynamics. Emotional contagion, as the term is used in social psychology, is generally defined as “the tendency to ‘catch’ (experience/express) another person’s emotions.”^74^ Or, in more words, “the process by which a person or group influences the emotions or behavior of another person or group through the conscious or unconscious induction of emotion states and behavioral attitudes.”^75^ Now, it is rather obvious that this definition refers to a psychological mechanism, not an affective experience. Emotional contagion does not denote an experience that anybody feels, but a mechanism which helps explaining how organisms affect each other.^76^

Over the last three decades, the leading paradigm in emotion research was the mimicry-based model of emotional contagion. Indeed, some suggest that it “has almost become a dogma in cognitive science.”^77^ Coincidentally, this is also the model that displays most similarities to the crowd psychological framework. In their seminal work, Hatfield, Cacioppo, and Rapson distinguish between a primitive and a more demanding, higher-level form of emotional contagion.^78^ They explain the primitive form with help of a two-step model: As the first step, individuals are taken to automatically mimic facial, vocal, and postural expressions; as a second step, an individual’s emotional experience is said to be affected by feedback from facial, vocal, and postural mimicry. Through this two-step process, individuals are considered to “catch” each other’s emotions and emotionally converge.^79^ Thus, this model suggests that emotional contagion is based on what Chartrand and Bargh have called the *chameleon effect*, that is the “nonconscious mimicry of the postures, mannerisms, facial expressions, and other behaviour of one’s interaction partners.”^80^ This conceptualization of primitive emotional contagion resembles the way in which the term was coined by Le Bon. It is characterized as “the tendency to automatically mimic and synchronize expressions, vocalizations, postures and movements with those of another person and, consequently, to converge emotionally.”^81^ In line with the crowd psychological framework, primitive emotional contagion is taken to be “relatively automatic, unintentional, uncontrollable, and largely unconscious.”^82^

Over the last few years, the mimicry-based model has been seriously challenged, both regarding its theoretical plausibility and its empirical support. Two challenges are particularly powerful. First, it seems unlikely that perceiving an emotion always elicits the same emotion in the perceiver. For instance, “the perception of another’s display of anger is likely to trigger fear and submission, not anger.”^83^ This challenges the plausibility of the key explanatory mechanism of the mimicry-based model. Maybe there are some cases of emotion transfer that go hand in hand with mimicry, but it does not seem plausible that this is always the case. Second, empirical findings “strongly suggest that emotional mimicry depends on a prior implicit interpretation process rather than direct matching of movements.”^84^ This challenges the view that processes through which someone “catches” the emotions of others are largely unconscious, automatic, and uncontrollable. By contrast, it seems more plausible to understand them as connected to a receiver’s appraisal of a sender’s emotional expression.^85^ Translated into phenomenological vocabulary, this suggests that contagion does in fact involve (at least a minimal) apprehension of the other’s emotion. This would imply that individuals can be aware of “catching” others’ emotions and that such processes are accompanied by control mechanisms that allow one to monitor and influence the process, at least to some extent. Finally, there might be cases in which emotions spread unconsciously and against an individual’s will, but in other instances such spreading is willingly allowed or even actively pursued.^86^ This last claim is supported, for instance, by recent research into affective scaffolding, which refers to manipulations of the environment aiming at regulating one’s own emotions.^87^

Much more could be said about this. But allow me to formulate the following tentative conclusion: On the one hand, the analogy with disease transmission seems to be the key ingredient constituting the persuasiveness of the crowd psychological framework. One might even hypothesize that this analogy is a major factor for the long-lasting acceptance of the mimicry-based model of emotional contagion. On the other hand, the disease model is the core of what is systematically wrong with the crowd psychological framework. Upon closer inspection, it is quite implausible that emotions spread like viruses. Virus infection happens without possible awareness and beyond the possibility of conscious control mechanism. As the Covid-19 pandemic has forcefully shown, it is impossible to notice the moment one is infected, or to resist infection through an act of the will. Only retrospectively one can try to trace the time and source of the infection (through measures like contact tracing). But this is not how emotional spreading works: It might be the case that we often do not notice how our moods and emotions are shaped by the environment. It might even be the case that we are secretly manipulated. And it might take much effort and retrospective reflection to find out about the manipulation and its source. However, our emotional lives are open to such reflection and emotional spreading is not in principle beyond possible awareness and control. All of this suggests that disease transmission is a bad analogy for a proper understanding of interpersonal emotion dynamics.

## Conclusion: Moving beyond the disease model of emotion transfer

In this paper, I have traced the influence of the crowd psychological framework within phenomenology, sociology, and social psychology. I have done so by examining paradigmatic accounts of key thinkers in each discipline. Moreover, I have shown that the persuasiveness of the crowd psychological framework relies on the analogies of the crowd with a herd of wild animals and of contagion with disease transmission. Now, what recommendation for future research does this paper provide? First, I suggest studying social gatherings free from the imagery of the crowd. This suggestion is neither new nor revolutionary, but still worth reemphasizing.^88^ Second, I recommend getting rid of the disease metaphor in social and cognitive science research on interpersonal emotion dynamics and in philosophical (and in particular phenomenological) investigations into affective we-experience. One step might simply be to stop using the term “contagion,” instead using less charged terms like “transfer,” “convergence,” “entrainment,” or even “effervescence.” Approaches to situated, embodied, and extended affectivity, or the theory of interaction rituals provide highly successful frameworks for studying emotional dynamics in gatherings of various sizes, so there is just no need to continue adhering to the problematic heritage of crowd psychology.^89^

## Footnotes

1 McPhail (1991); Borch (2012).

2 Taine (1876).

3 The following remarks about Taine’s book are based on Borch (2012, pp. 28–32).

4 Le Bon (1895). The English translation of this text is Le Bon (1896a). Le Bon (1894). The English translation of this text is Le Bon (1898). Le Bon (1896b). The English translation of this text is Le Bon (1899).

5 Le Bon (1895, p. i).

6 Le Bon (1895, p. v).

7 Le Bon (1895, p. i; 1896a, p. v) The English edition translates “l’âme des foules” as “characteristics of crowds” and “l’âme de cette race” as “the genius of the race.”

8 Le Bon’s racism is firmly connected with his elitism, as the following sentence from the introduction to *The Psychology of Peoples* exemplifies: “There is no psychologist, no traveller [sic!], no fairly intelligent statesman who is not aware how erroneous is this chimerical notion of the equality of men, which has thrown the world into confusion, brought about in Europe a gigantic revolution, involved America in the sanguinary War of Succession and landed all the French colonies in a state of lamentable decadence.” Le Bon (1898, pp. xv–xvi).

9 Le Bon (1896a, p. v).

10 Le Bon (1913, p. 104).

11 Thonhauser (2020a).

12 Le Bon (1896a, p. xix).

13 Le Bon (1896a, p. 2).

14 Le Bon (1913, p. 102).

15 Le Bon (1896a, p. 9).

16 Le Bon (1896a, p. 13). In a later text, Le Bon writes almost identically: “These various characteristics [of the crowd] show that man in the crowd descends to a very low degree in the scale of civilization.” Le Bon (1913, p. 103.)

17 Le Bon (1896a, p. 10).

18 Le Bon (1896a, p. 10).

19 Le Bon (1896a, p. 11.)

20 Tarde (1890).

21 Tarde (1903).

22 Le Bon (1908).

23 Tarde (2003).

24 Tarde (1903, pp. ix–x).

25 Tarde (1903, pp. xvii).

26 Borch (2012, pp. 48–78). Only recently Tarde has been rediscovered, most importantly in connection with the works of Bruno Latour and of Gilles Deleuze and Félix Guattari. See Candea (2010).

27 Durkheim (1894). To my knowledge, the first English edition is Durkheim (1938).

28 Durkheim (1964, p. 215f).

29 Durkheim work is based on ethnological studies of religious ceremonies of indigenous Australians.

30 Simmel (1908). To my knowledge, Simmel’s book, at least in its entirety, was first translated into English 100 years later. Simmel (2009).

31 Simmel (2009, p. 20).

32 Simmel (2009, p. 22).

33 Simmel (2009, p. 58). The translators of Simmel’s sociology decided to translate “*Masse*” as “mass.” However, “crowd” would be equally valid as a translation, as the German language knows only the term “*Masse*.”

34 Weber (1922; English translation 1978).

35 Weber (1978, p. 3).

36 Weber (1978, p. 23).

37 Weber (1978, pp. 23–24).

38 Thonhauser and Wetzels (2019).

39 Tönnies (1887).

40 Scheler (2009, pp. 8–18).

41 Schmid (2008); Konzelmann Ziv (2009); Salice (2015); Schloßberger (2016).

42 Scheler (1913a).

43 Scheler (1913b; 1916).

44 Scheler (1923).

45 Scheler (1973, p. 526).

46 Scheler (2009, p. 35).

47 Scheler (1913a, p. 12).

48 Freud (1921).

49 Le Bon (1912). The book was translated into English the following year. Le Bon (1913). To my knowledge, this book has not been translated into German. I was informed by an anonymous reviewer that a review of the book *La Révolution Française et la Psychologie des Révolutions* with the title “*L’âme révolutionnaire*” was published in a French periodical. Gaultier (1912).

50 Le Bon (1913, pp. 102–105).

51 Scheler (2009, p. 8).

52 Scheler uses the term “*Nachfühlen*” which is difficult to translate into English: It means apprehending or understanding via feeling; in other words: it indicates that the feeling does the apprehending or understanding of the experiences of others.

53 Scheler (2009, p. 12; 1913a, p. 9). It is somewhat confusing that Scheler uses the term fellow-feeling as a heading for the entire taxonomy and as a subcategory.

54 Scheler (2009, pp. 18–36; 1923, pp. 16–40).

55 Scheler (2009, p. 13; 1913a, p. 9).

56 For a defense of the token-identity claim see Schmid (2008). For alternative readings see Krebs (2010); Salice (2015); Schloßberger (2016).

57 Scheler (2009, p. 13; 1913a, p. 9).

58 Scheler (2009, p. 14f; 1913a, p. 10).

59 Scheler (2009, p. 15f; 1913a, p. 12).

60 Scheler (2009, p. 15; 1913a, p. 11).

61 Scheler (2009, p. 17; 1913a, p. 13).

62 Scheler (2009, p. 15; 1913a, p. 11).

63 Le Bon (1913, e.g., p. 12, p. 36 and p. 106).

64 Stein (2000, p. 244).

65 Stein (2000, p. 244).

66 Zahavi (2015, p. 87).

67 Scheler (2009, p. 13; 1913a, p. 9).

68 Scheler (2009, p. 15; 1913a, p. 11).

69 Scheler (1982, pp. 272–282.

70 Scheler (2009, p. 36).

71 As a further case in point, the examples of emotional contagion which Scheler provides in *The Nature of Sympathy* are motivated by sexist prejudices: He writes that laughter is particularly contagious in female children. Similarly, he asserts that older women are especially susceptive to lamentation. Scheler (2009, p. 15; 1913a, p. 11).

72 Thonhauser (2022).

73 Scheler (2009, p. 17; 1913a, p. 13).

74 Hatfield et al. (1992, p. 153).

75 Schoenewolf (1990, p. 50).

76 Thonhauser (2022).

77 Dezecache et al. (2015, p. 297).

78 Hatfield et al. (1994).

79 Hatfield et al. (2009, pp. 19–31).

80 Chartrand and Bargh (1999, p. 893).

81 Hatfield et al. (1992, pp. 153–154).

82 Hatfield et al. (1992, p. 153).

83 Dezecache et al. (2015, p. 298).

84 Parkinson (2020, p. 3).

85 Dezecache et al. (2016, pp. 417–438); Isern-Mas and Gomila (2019, pp. 71–100).

86 Thonhauser (2020b).

87 Colombetti and Krueger (2015).

88 For similar recommendations see, e.g., McPhail (1991); Borch (2012).

89 Collins (2004), Fuchs (2013), Griffiths and Scarantino (2008), Krueger and Szanto (2016), Thonhauser (2021).
